# Effectiveness of fissure sealants in 8- to 10-year-olds with and without molar–incisor hypomineralization (MIH) – results from a cross-sectional epidemiological study

**DOI:** 10.1007/s00784-024-06083-6

**Published:** 2024-12-18

**Authors:** Felicitas Zöllner, Karl-Ferdinand Fresen, Ramy Gaballah, Helen Schill, Vinay Pitchika, Stefanie Amend, Norbert Krämer, Jan Kühnisch

**Affiliations:** 1 Department of Conservative Dentistry and Periodontology, University Hospital, Ludwig-Maximilians-Universität München, Munich, Germany; 2 Department of Paediatric Dentistry, Medical Centre for Dentistry, University Medical Center Gießen and Marburg, Justus-Liebig-Universität Gießen, Gießen, Germany; 3 Klinik für Zahnerhaltung und Parodontologie, Klinikum der Ludwig-Maximilians-Universität München, Goethestraße 70, 80336 München, Germany

**Keywords:** Dental caries, Fissure sealant, Enamel hypomineralization, Epidemiology, Cross-sectional study

## Abstract

**Objective:**

This cross-sectional study aimed to investigate the use, quality, and caries-preventive effects of fissure sealants (FSs) in 8- to 10-year-olds with and without molar–incisor hypomineralization (MIH).

**Materials and methods:**

A total of 5,418 children (2,692 males, 2,726 females) were examined via standard instruments (dental mirrors, CPI probes, adequate lighting, mobile examination tables, and air syringes) and methods for the recording of caries (DMFT index, WHO method) and FSs. The classification of MIH followed the recommendations of the European Academy of Pediatric Dentistry (EAPD). Statistical analysis included descriptive analysis and mixed-effects logistic regression models.

**Results:**

59.0% of all children had at least one FS; this percentage was 57.8% in the MIH group. The proportions of fully intact sealants and minimal, moderate, or nearly complete loss of retention were 31.7%, 48.3%, 16.8%, and 3.2%, respectively. The mean caries experience was low, at 0.2 DMFT in the whole population. Lower DMFT means were registered in individuals with FSs without MIH (0.1) and with MIH (0.1). The regression analysis revealed a significant caries-protective effect of FSs and MIH in relation to the overall caries burden. In addition, the caries-protective effect was greater in individuals with fully retained sealants (aOR 0.269) than in those with minimal (aOR 0.346), moderate (aOR 0.567) or nearly complete loss of retention (aOR 0.721).

**Conclusion:**

This study documented the comparable use, quality, and caries-preventive effects of FSs in individuals and permanent molar with and without MIH.

**Clinical relevance:**

FSs are caries protective in children with and without MIH.

## Introduction

Caries has declined in many industrialized nations due to the implementation of evidence-based measures to prevent and control the disease. In addition to being used for dietary and biofilm control, fissure sealants (FSs) are recommended to prevent or arrest caries on pits and fissures [1]. The effectiveness of FSs for caries prevention has been clearly shown in several systematic reviews and meta-analyses [2–6] as long as they are completely retained. With respect to the flowable material properties, which are linked to a reduced filler content, long-term survival is potentially reduced compared with that of other composite-based restoration materials. It can be estimated from systematic reviews and meta-analyses that, as a rule of thumb, ∼ 10% of resin-based sealants are potentially affected by material loss over one year of observation [7, 8]. Furthermore, long-term retention depends on the chosen material, the use of the acid etching technique, the individual processing of the FS application, and patient cooperation [6–9]. This highlights the fact that FS quality can be affected by multiple factors. When quality data from cross-sectional epidemiological studies are considered, retention is more severely affected [10, 11] than when the results of well-controlled clinical trials are examined. Therefore, it can be assumed that cross-sectional studies provide more realistic data about population-based FS use and retention. Consequently, the documentation of FSs and their quality should be included in epidemiological studies and investigated regularly.

Enamel hypomineralization in terms of molar–incisor hypomineralization (MIH) has become a prevalent finding in recent decades [12]. Unfortunately, little is known about the clinical usefulness and effectiveness of sealing hypomineralized permanent molars. Only a few clinical studies aimed at exploring divergent outcomes have been published [13–15]. In addition, FSs are inconsistently recommended for MIH children [16–18], which potentially results in an uncertain use of FSs on hypomineralized molars in daily dental practice. The reluctant use of FSs might be attributed primarily to their unpredictable adhesion to hypomineralized enamel, which potentially results in higher rates of material loss than healthy enamel [19, 20]. Moreover, FSs are also used on permanent molars affected by MIH in dental practice; however, no information is available from cross-sectional studies regarding the level at which this caries-preventive measure is used in adolescents. Furthermore, no information is available about sealant quality in MIH molars. Given the knowledge gaps, this cross-sectional study aimed to register the use, quality, and effectiveness of FSs for caries prevention in a representative sample of 8- to 10-year-old children in Bavaria, Germany. The null hypothesis was that caries are equally distributed in children with and without sealants and with and without MIH, which is closely linked to the determination of the effectiveness of FSs in the investigated group of children.

## Materials and methods

This cross-sectional epidemiological study in 8- to 10-year-old Bavarian primary schoolchildren received approval from the Ethics Commission at the Justus-Liebig-University Gießen (AZ 72/22) and was conducted by two study centres (North Bavaria: University of Gießen; South Bavaria: LMU of Munich) in close cooperation with the Bavarian workgroup for public dental health (Bayerische Landesarbeitsgemeinschaft Zahngesundheit e.V., LAGZ). All examinations were conducted in compliance with the ethical standards of the Institutional Review Board (IRB) and the modified Helsinki Declaration [21]. The legal guardians had to hand in written consent. The reporting recommendations of the STROBE guidelines for observational studies were followed [22].

### Sample size and recruitment of the study population

Before initiating this epidemiological study, the sample size was estimated according to the population-based data published by the Bavarian Statistics Office and experiences from previously conducted dental health surveys in the same setting [23]. To ensure a precision of no more than 2.1% in estimating the prevalence of caries and MIH, a sample size of 5,000 individuals was deemed necessary. Thus, an initial sample size of 6,500 pupils from 76 schools, covering all 8–10-year-olds in the 3rd and 4th grades, was set. Unfortunately, 16 schools refused their participation after the invitation, which required additional recruitment later. In parallel, a low participation rate (< 50%) was recognized during the first school examinations. Therefore, additional recruitment was planned, which included another 40 randomly selected schools. The final dataset included dental health information from 5,418 primary schoolchildren (mean 9.6 years, 2,692 males, 2,726 females, and 87 primary schools). The overall participation rate was 50.4%.

### Dental examinations

All dental examinations were performed from March until July 2023. Trained and calibrated dentists conducted all the clinical examinations in the school setting. Each schoolchild underwent an examination during a designated school appointment on a mobile patient examination table (PINO mobile massage bed “Atoll”, Pino Pharmazeutische Präparate, Hamburg, Germany) or a regular school chair. Adequate lighting was provided throughout all the examinations via either a halogen lamp (Haeberle Halux 50 S, 50 W, Haeberle GmbH, Stuttgart, Germany) or a high-power headlamp. Standardized dental examination tools included a mirror and a blunt CPI probe (CP-11.5B6, Hu-Friedy, Chicago, IL, USA). Before the dental status was documented, each tooth surface was dried via an air syringe powered by a portable compressor (SNR MAC-A-002670, DTS Design, Mammendorf, Germany). In the case of below-average oral hygiene, dental plaque was removed either by using cotton rolls or by advising the individual to brush their teeth.

### Scoring of caries, MIH and sealants

The examination documented the status of all available primary and permanent teeth. Caries, MIH, and FSs were recorded independently from each other, and the documentation form allowed the recording of multiple findings per tooth surface. The caries condition was assessed via the dmf/t and DMF/T indices according to the WHO protocol [24]. A caries lesion (d/t and D/T) was documented when a tooth was clearly cavitated, showed undermined enamel, or softening of dental tissue was detected [24]. Noncavitated caries lesions (i/t and I/T) were evaluated on the basis of the ICDAS/UniViSS criteria [25, 26] and were summarized in one score. The classification of MIH-related findings followed the recommendations of the European Academy of Pediatric Dentistry (EAPD). Here, the following criteria for classification were used: (1) demonstrated opacity > 2 mm, (2) enamel breakdown, (3) atypical restoration, (4) extraction, and (5) indirect restoration due to MIH [16, 18]. Other developmental defects, such as fluorosis, amelogenesis imperfecta, or dentinogenesis imperfecta, were not scored and, therefore, were not considered. The retention of FSs was scored according to the following scores: (1) occlusal surface with intact FSs (loss of material up to one-third at the periphery of the fissure pattern), (2) minimal loss of retention exclusively at the periphery of the occlusal surface, (3) occlusal surface with sufficient FSs (retention of the material in the main fissure or loss of material exceeding one-third of the fissure pattern), and (4) insufficient FSs (nearly complete loss of material and re-exposure of the main fissures) [27]. The type of sealant material was not scored. Furthermore, the clinical use of sealants or other dental interventions was not counterchecked by interviewing the children or their parents/legal guardians.

### Calibration of the study team

Before beginning the field investigations, all examiners underwent three days of calibration training led by experienced oral epidemiologists. The workshop aimed to determine how to score cavitated and noncavitated carious lesions, MIH, and FSs. The theoretical component covered the study design, indices, and diagnostic principles, whereas the practical aspect involved analysing and discussing various high-quality photographs of individual tooth surfaces depicting carious lesions, MIH, FSs, and potential differential diagnoses. Following the calibration training, each examiner assessed 60 unknown, high-quality photographs of occlusal and buccal/palatal or lingual surfaces, aiming to detect caries, MIH, and FSs. This evaluation was repeated approximately four weeks later. Inter- and intra-examiner kappa values were calculated to establish reliability, with interpretations based on the following criteria: 0.0 to 0.2 – slight agreement, 0.21 to 0.40 – fair agreement, 0.41 to 0.60 – moderate agreement, 0.61 to 0.80 – substantial agreement, and 0.81 to 1.00 – (almost) perfect agreement. The intra-examiner kappa values for detecting caries, MIH, and FSs were in moderate to perfect agreement for all examiners (caries: 0.697 to 1.000, MIH: 0.484 to 1.000, sealants: 0.726 to 1.000). The inter-examiner reliabilities of the examiners varied between fair and perfect agreement (caries: 0.550 to 0.949, MIH: 0.371 to 0.956, sealants: 0.602 to 1.000).

### Statistical analysis

The collected data were collected via a customized database (EpiData - Comprehensive Data Management and Basic Statistical Analysis System. Odense Denmark, EpiData Association, 2010; http://www.epidata.dk) and later imported into an Excel spreadsheet (Excel 2019, Microsoft Corporation, Redmond, WA, USA). Microsoft Excel 365 and the statistical software R (http://www.r-projekt.org, version 4.3.1) were used to compare the sets of data. The descriptive statistical analysis included the calculation of caries and MIH prevalence rates as well as the determination of the mean number and standard deviation of caries-affected teeth. Caries experience in children with or without sealants in relation to different thresholds (children with or without MIH) was determined, and the mean tooth number (standard deviation) was calculated for each category. Additionally, descriptive statistical analysis addressed the description of sealant quality in relation to the presence of caries and MIH.

Owing to the structure of the data, i.e., multiple teeth (permanent first molars) per patient, the data from its wide format (each row representing a patient) were converted into a long format (each row corresponding to a first permanent molar (FPM), resulting in 4 rows per patient), and an index variable (tooth number) was generated. Mixed-effects logistic regression models were constructed for two outcomes: (i) IDMF = 0 vs. IDMF > 0 and (ii) full retention/minimal loss of retention of fissure sealant vs. moderate/nearly complete loss of retention. Both models were adjusted for covariates such as age, sex, region, and MIH (FPM). Additionally, the level of fissure sealant retention was included as a covariate in the caries burden model, and the caries status was included as a covariate in the sealant quality model. Furthermore, to account for the presence of multiple teeth within the same patient, the tooth number variable was included as a random effect in both models. Model diagnostics, including checks for multicollinearity, assessment of residuals, and evaluation of random effect assumptions, were performed to ensure the appropriateness of the models. All the statistical analyses were performed via Stata/MP 18.0 (StataCorp 2023, College Station, TX, USA). A *p*-value less than 0.05 was considered statistically significant; hence, the null hypothesis should be rejected. A *p*-value above 0.05 means that the null hypothesis can be accepted.

## Results

**Fig. 1:**
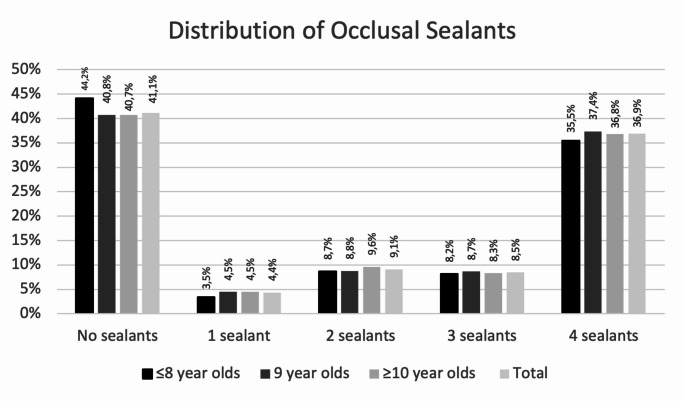
Distribution of fissure sealants in 8- to 10-year-olds from Bavaria, Germany

In this cross-sectional study, 5,418 Bavarian 8- to 10-year-old primary schoolchildren were examined. A total of 11.1% (DMF > 0) and 24.3% (IDMF > 0) of the entire study population were scored as having caries, and 17.5% were affected by MIH (Table 1). A total of 59.0% (*N* = 3,197) of all the children had at least one FS; this percentage was 57.8% (*N* = 547) in the MIH group (Table 1; Fig. 1). The proportion of caries-free individuals (DMF = 0) was slightly greater in children with sealants (without MIH: 91.4%; with MIH: 92.9%) than in those without sealants (without MIH: 84.8%; with MIH: 86.0%). The mean number of caries experiences can be taken from Table 2. In detail, the mean DMFT/IDMFT value was low in the whole population, at 0.3/0.6 (Table 2). Lower DMFT/IDMFT means were registered in individuals with FSs without MIH (0.1/0.4) and with MIH (0.1/0.2) (Table 2). The number of sealed molars per individual is illustrated in Fig. 1.

**Table 1:** Prevalence rates of caries in 8- to 10-year-old children with and without sealants from Bavaria, Germany

Caries prevalence	Children with sealants (*N* = 3,197; 59.0%)				Children without sealants (*N* = 2,221; 41.0%)				All Children (*N* = 5,418; 100.0%)	
	Children with MIH (*N* = 547; 10.1%)		Children without MIH (*N* = 2,650; 48.9%)		Children with MIH (*N* = 399; 7.4%)		Children without MIH (*N* = 1,822; 33.6%)			
	*N*	%	*N*	%	*N*	%	*N*	%	*N*	%
dmf = 0	325	59.4	1,515	57.2	249	62.4	1,115	61.2	3,204	59.1
idmf = 0	305	55.8	1,370	51.7	223	55.9	993	54.5	2,891	53.4
DMF = 0	508	92.9	2,421	91.4	343	86.0	1,545	84.8	4,817	88.9
IDMF = 0	471	86.1	2,113	79.7	299	74.9	1,218	66.8	4,101	75.7
dmf/DMF = 0	313	57.2	1,437	54.2	227	56.9	1,021	56.0	2,998	55.3
idmf/IDMF = 0	273	49.9	1,193	45.0	193	48.4	785	43.1	2,444	45.1

**Table 2:** Mean tooth numbers and standard deviations for caries in relation to the presence of sealants and MIH in 8- to 10-year-olds from Bavaria, Germany

Caries experience	Children with sealants (*N* = 3,197; 59.0%)				Children without sealants (*N* = 2,221; 41.0%)				All Children (*N* = 5,418; 100.0%)	
	Children with MIH (*N* = 547; 10.1%)		Children without MIH (*N* = 2,650; 48.9%)		Children with MIH (*N* = 399; 7.4%)		Children without MIH (*N* = 1,822; 33.6%)			
	Mean	SD	Mean	SD	Mean	SD	Mean	SD	Mean	SD
i/t	0.2	0.6	0.2	0.7	0.2	0.7	0.3	0.8	0.2	0.7
d/t	0.2	0.6	0.3	0.8	0.4	1.0	0.5	1.1	0.3	0.9
m/t	0.0	0.3	0.1	0.3	0.0	0.2	0.1	0.4	0.1	0.4
f/t	1.1	2.0	1.2	2.0	0.8	1.6	0.8	1.6	1.0	1.9
dmf/t	**1.3**	**2.1**	**1.5**	**2.3**	**1.2**	**2.0**	**1.3**	**2.2**	**1.4**	**2.2**
idmf/t	**1.5**	**2.2**	**1.7**	**2.4**	**1.4**	**2.0**	**1.6**	**2.3**	**1.6**	**2.3**
I/T	0.1	0.5	0.3	0.8	0.4	1.2	0.6	1.3	0.4	1.0
D/T	0.0	0.2	0.0	0.2	0.1	0.4	0.1	0.5	0.1	0.3
M/T	0.0	0.0	0.0	0.0	0.0	0.0	0.0	0.1	0.0	0.1
F/T	0.1	0.4	0.1	0.4	0.2	0.6	0.3	0.8	0.2	0.6
DMF/T	**0.1**	**0.4**	**0.1**	**0.5**	**0.3**	**0.8**	**0.4**	**1.0**	**0.3**	**0.7**
IDMF/T	**0.2**	**0.7**	**0.4**	**1.0**	**0.7**	**1.5**	**1.0**	**1.6**	**0.6**	**1.3**

However, another aspect to examine was the sealant distribution and quality on all first permanent molars in relation to the presence of caries-related findings (Table 3). In general, 49.1% (*N* = 10,603) of all first permanent molars were sealed; in contrast, 50.9% (*N* = 10,969) remained free of FSs. The proportion of fully intact sealants was 31.7% (*N* = 3,366). Minimal loss of retention was found in 48.3% (*N* = 5,119) of all the sealed permanent molars. Clinical signs of insufficiency were detected in 20.0% of all sealants (16.8% (*N* = 1778) moderate loss of retention and 3.2% (*N* = 340) nearly complete loss of retention). Noncavitated (1.5%; *N* = 154) or cavitated caries lesions (0.1%; *N* = 8) were rarely scored in sealed molars. In contrast, noncavitated caries (10.6%; *N* = 1,168) and cavitated caries (1.5%; *N* = 168) were more frequently detected in unsealed molar teeth.

**Table 3:** Sealant quality of occlusal fissures in relation to the presence of caries and caries-related restorations on first permanent molars in 8- to 10-year-olds from Bavaria, Germany

Sealant quality	Healthy		Noncavitated caries		Cavitated caries		Filled		Total	
	*N*	%	*N*	%	*N*	%	*N*	%	*N*	%
Unsealed	8,880	41.2	1,168	5.4	168	0.8	753	3.5	10,969	50.9
Full retention	3,362	15.6	1	0.0	1	0.0	2	0.0	3,366	15.6
Minimal loss of retention	5,099	23.6	18	0.1	1	0.0	1	0.0	5,119	23.7
Moderate loss of retention	1,672	7.7	100	0.5	2	0.0	4	0.0	1,778	8.2
Nearly complete loss of retention	301	1.4	35	0.2	4	0.0	0	0	340	1.6
Total	19,314	89.5	1,322	6.2	176	0.8	760	3.5	21,572*	100.0

*100 first permanent molars were unerupted, extracted, missing or not assessable due to orthodontic applications or cooperation

The distribution and quality of FSs were also analysed on first permanent molars affected by MIH (Table 4). A total of 9.3% (*N* = 2,008) were diagnosed as having at least one MIH finding out of all the molar teeth (*N* = 21,556). Demarcated opacities (*N* = 1,282) were most frequently detected, followed by enamel breakdowns (*N* = 431) and atypical restorations (*N* = 295). The proportions of sealed/unsealed molars were as follows: 636/646 (demarcated opacity), 126/305 (enamel breakdowns), and 27/268 (atypical restorations). A total of 39.3% (*N* = 789) of all first permanent molars with MIH (*N* = 2,008) were sealed. When the descriptive data for sealant quality in first permanent molars with MIH were analysed, the following percentages were calculated from Table 4: 34.1% were fully intact sealants (*N* = 269), 46.9% were minimally lost sealants (*N* = 370), 16.7% were moderately lost sealants (*N* = 132), and 2.3% were nearly completely lost sealants (*N* = 18).

**Table 4:** Sealant quality of occlusal fissures in relation to MIH-related findings on first permanent molars in 8- to 10-year-olds from Bavaria, Germany

Sealant quality	Healthy		Demarcated opacities		Enamel breakdown		Atypical restoration		Total	
	*N*	%	*N*	%	*N*	%	*N*	%	*N*	%
Unsealed	9,734	45.2	646	3.0	305	1.4	268	1.2	10,953	50.8
Full retention	3,097	14.4	238	1.1	20	0.1	11	0.1	3,366	15.7
Minimal loss of retention	4,749	22.0	290	1.4	71	0.3	9	0.0	5,119	23.7
Moderate loss of retention	1,646	7.6	90	0.4	35	0.2	7	0.0	1,778	8.2
Nearly complete loss of retention	322	1.5	18	0.1	0	0	0	0	340	1.6
Total	19,548	90.7	1,282	6.0	431	2.0	295	1.3	21,556*	100.0

*116 first permanent molars were unerupted, extracted, missing or not assessable due to orthodontic applications or cooperation

The data were explored statistically via two different mixed-effects logistic regression models (Tables 5 and 6). In the first model, the influences of MIH and FSs were estimated in relation to the overall caries burden (IDMF) in the permanent dentition (Table 5). Both variables had a significant caries-protective effect. The effect of sealant quality should be highlighted, as the effectiveness of fully retained sealants (aOR 0.269) was greater than that of sealants with minimal (aOR 0.346), moderate (aOR 0.567), or complete loss of retention (aOR 0.721). When the influence of enamel hypomineralization and caries on sealant quality was considered, significant associations were observed (Table 6). Enamel breakdowns (aOR 1.630), noncavitated caries lesions (aOR 31.407), cavitated caries lesions (aOR 11.362), and caries-related restorations (aOR 5.538) were more likely to be associated with moderate or nearly complete retention losses (Table 6). Notably, age, sex and region significantly coinfluenced both models (Tables 5 and 6).

**Table 5:** Distribution of exposure (fissure sealant quality) and covariates and adjusted odds ratios (aORs) from the mixed-effects logistic regression model for IDMF = 0 vs. IDMF > 0 in relation to fissure sealant quality, adjusted for age, sex, region, and MIH diagnosis of first permanent molars. To adjust the multilevel nature of the dataset, the tooth number (four first permanent molars) was included as a random effect

		Case distribution				Mixed-effects logistic regression model	
		IDMF = 0		IDMF > 0		IDMF = 0 versus IDMF > 0	
						aOR (95% CI)	*p*-value
Age	Mean ± standard deviation	9.3 ± 0.7		9.5 ± 0.8		**1.408 (1.348–1.471)**	**< 0.0001**
Sex	Female (*N*/%)	8,216	37.9	2,688	12.4	-	-
	Male (*N*/%)	8,188	37.8	2,580	11.9	**0.926 (0.868–0.987)**	**0.019**
Region	Urban (*N*/%)	5,124	23.6	1,580	7.3	-	-
	Rural (*N*/%)	11,280	52.1	3,688	17.0	1.005 (0.937–1.078)	0.889
MIH diagnosis on first permanent molars	Healthy (*N*/%)	14,636	67.9	4,912	22.8	-	-
	Demarcated opacity (*N*/%)	1,084	5.1	198	0.9	**0.525 (0.448–0.626)**	**< 0.0001**
	Enamel breakdown (*N*/%)	375	1.7	56	0.3	**0.352 (0.264–0.468)**	**< 0.0001**
	Atypical restoration (*N*/%)	224	1.0	71	0.3	**0.628 (0.478–0.825)**	**0.001**
Fissure sealants	Unsealed (*N*/%)	7,382	34.2	3,592	16.7	-	-
	Full retention (*N*/%)	2,964	13.7	402	1.9	**0.269 (0.240–0.301)**	**< 0.0001**
	Minimal loss (*N*/%)	4,361	20.2	758	3.5	**0.346 (0.317–0.378)**	**< 0.0001**
	Moderate loss (*N*/%)	1,380	6.4	398	1.8	**0.567 (0.503–0.639)**	**< 0.0001**
	Nearly complete loss (*N*/%)	250	1.2	90	0.4	**0.721 (0.564–0.922)**	**0.009**

**Table 6:** Distribution of exposure (fissure sealant quality) and covariates and adjusted odds ratios (aORs) from the mixed-effects logistic regression model for fissure sealant retention in relation to caries status, adjusted for age, sex, region and MIH diagnosis of first permanent molars. To adjust the multilevel nature of the dataset, the tooth number (four first permanent molars) was included as a random effect

		Case distribution				Mixed-effects logistic regression model	
		Full retention/minimal loss of retention		Moderate/nearly complete loss of retention		Full retention/minimal loss versus Moderate/nearly complete loss	
						aOR (95% CI)	*p*-value
Age	Mean ± standard deviation	9.3 ± 0.7		9.4 ± 0.7		**1.110 (1.036–1.190)**	**0.003**
Sex	Female (*N*/%)	4,361	41.1	1,002	9.5	-	-
	Male (*N*/%)	4,124	38.9	1,116	10.5	**1.160 (1.051–1.280)**	**0.003**
Region	Urban (*N*/%)	2,904	27.4	589	5.6	-	-
	Rural (*N*/%)	5,581	52.6	1,529	14.4	**1.347 (1.209–1.502)**	**< 0.0001**
MIH diagnosis on first permanent molars	Healthy (*N*/%)	7,846	74.0	1,968	18.5	-	-
	Demarcated opacity (*N*/%)	528	5.0	108	1.0	0.841 (0.678–1.045)	0.118
	Enamel breakdown (*N*/%)	91	0.9	35	0.3	**1.630 (1.095–2.426)**	**0.016**
	Atypical restoration (*N*/%)	20	0.2	7	0.1	1.642 (0.687–3.924)	0.265
Caries	Healthy (*N*/%)	8,461	79.8	1,973	18.6	-	-
	Noncavitated caries (*N*/%)	19	0.2	135	1.3	**31.407 (19.314–51.073)**	**< 0.0001**
	Cavitated caries (*N*/%)	2	0.0	6	0.1	**11.362 (2.258–57.171)**	**0.003**
	Filling (*N*/%)	3	0.0	4	0.0	**5.538 (1.217–25.199)**	**0.027**

## Discussion

This cross-sectional epidemiological study examined the clinical use, quality and effectiveness of FSs in 8- to 10-year-olds with and without MIH. The main results revealed that the incidence of caries was lower in children with FSs than in children who did not receive this preventive measure (Tables 1, 2, 4 and 5); this is true for both children with and without MIH. Therefore, the initially formulated hypothesis must be rejected, as there was not an equal distribution of caries among children with and without MIH and with and without FSs. In addition, sufficient FS retention was associated with a reduced probability of caries in comparison with insufficient FS retention (Table 5).

In the study population, 59.0% of all 8- to 10-year-olds had at least one FS (Table 1), with the majority of individuals having all four first permanent molars sealed (Fig. 1). Compared with previous epidemiological surveys in Germany [10, 11], the use of FSs has been largely consistent over the past two decades. This finding indicates that pit and fissure sealing has been an established preventive measure since its introduction in 1996 and is currently consistently used by the dental profession. In Germany, this is supported by the fact that FSs are used in preventive dental health care programs for children aged up to 17 years and are therefore available in this population. Notably, the quality of FSs can also be considered satisfactory. A total of 80.1% of all FSs were either completely intact (*N* = 3,366; 31.7%) or, at most, showed a minimal loss of retention (*N* = 5,119; 48.3%). If this order of magnitude is set in relation to previously published quality data in Germany, significantly lower proportions of intact sealants were sometimes published in epidemiological studies in the past [28]. However, notably, recording the quality of FSs in epidemiological studies has rarely been the subject of investigations.

A significant caries-preventive effect of FSs in young permanent dentition was shown descriptively (Tables 1, 2, 4 and 5) as well as in the mixed-effects logistic regression model (Table 5). Interestingly, sealant effectiveness increased with the degree of sealant intactness (Table 5), which supports the assumption that sealants can be effective in preventing pit and fissure caries as long as they are completely retained (Table 5). The probability of a lack of sealant quality increased in cases of noncavitated/cavitated carious lesions and MIH-related enamel breakdown (Table 6). In the case of MIH-related hard tissue defects, mainly the enamel structure and its impact on the adhesive compound must be reasonably discussed. A loss of material in relation to caries can be discussed in two ways. First, sealant loss may have occurred after occlusal caries were arrested by sealing due to an imperfect adhesive compound. A second option might be that re-exposed pits and fissures develop caries due to increased caries activity. However, the preventive effect of caries is extensively documented and comprehensively illustrated in representative epidemiological studies in Germany [10, 29] as well as in systematic reviews/meta-analyses [2, 30, 31], which is why further discussion can be refrained from at this point. The negative associations between MIH-related opacities, enamel breakdowns and atypical restorations and the presence of caries can also be seen in Table 5. This finding is in line with previous studies and was extensively discussed in a separate analysis [32]. When age is considered an influencing variable, it must be referred to as the spectrum of age in the study population—from 8 to 10 years—and the common finding that caries experience increases with increasing age. This common trend explains the documented adjusted odds ratio (Table 5).

The effectiveness of FSs as a caries-preventive measure in relation to the presence of MIH was analysed for the first time. In principle, an MIH incidence of 17.5% was documented for the population studied (Tables 1 and 2), which aligns with the literature and published systematic reviews/meta-analyses [12, 33]. By recording MIH, FSs, and caries separately, it was possible to determine MIH statistically in relation to the presence of FSs and caries. Here, both the descriptive (Tables 1 and 2, and 4) and explorative (Tables 5 and 6) data indicate a caries-protective effect of FSs, as the caries prevalence and experience were significantly lower in adolescents with and without MIH. While the caries-protective effect of FSs has been clearly described in the literature [2, 30, 31, 34], no epidemiological data on the benefits of FSs in adolescents with MIH have been identified to date. Owing to the lack of methodologically identical studies, further discussion is impossible at this time. However, the prevalence and experience of caries documented in this study were lower in the MIH group than in the other groups. The present finding—of a lower caries burden among MIH patients—appears to be consistent with previous studies in similar settings [35, 36]. However, these results (Table 2) contradict published systematic reviews/meta-analyses [37–40], which documented an increased caries burden for MIH patients. The reason for this discrepancy is that, on the one hand, a higher socioeconomic status has been demonstrated for children and adolescents with MIH [41], which is typically associated with lower caries rates. This trend may be supported by the use of FSs (Table 2). On the other hand, the methodology of independent recording of caries and MIH should be emphasized, as it is associated with a clear classification of dental hard tissue defects and restorations and potentially prevents misclassifications. In addition, it should be noted that a few existing clinical studies involving MIH patients documented positive effects of FSs [42, 43]. Furthermore, studies have reported a reduction in the hypersensitivities of teeth affected by MIH [15].

With respect to the frequency and quality of the FSs, 59.0% of all the examined individuals had at least one FS (Tables 1 and 2). This rate was almost identical for children with MIH at 57.8% (*N* = 547/946), which means that FSs were used approximately equally in the groups of children with or without MIH. Tables 1 and 2 show that FS use has an additional caries-preventive effect on MIH individuals compared with individuals without FSs. Interestingly, the quality of FSs in MIH patients did not differ substantially from that in non-MIH individuals. However, this finding requires further investigation, as no information was collected about the exact location of the MIH molars. Hypomineralization may have a substantial impact if it is located directly in the pit and fissure pattern or outside of the pit or fissure pattern on vestibular or oral surfaces that cannot be sealed. Hypothetically, it can be assumed that the sealing of hypomineralized enamel is potentially linked to higher rates of retention losses due to diminished adhesion compared with regular enamel bonding and due to its long-term survival [44]. However, epidemiological or clinical data that analyse FS retention in relation to the MIH pattern are not available, so further investigations with more sophisticated study designs are needed.

This epidemiological cross-sectional study has both strengths and limitations. The methodological strengths include the examination of 5,418 primary schoolchildren and the separate recording of caries, MIH, and FSs in the entire dentition. Moreover, the population studied had a low caries prevalence and experience, which means that MIH and FSs could be recorded largely without distortion. The limited participation rates of schools and individuals should be recognized as a significant limitation. While the participation rate at the school level was 75.0%, the overall participation rate was only 50.4%. This affects the representativeness of this study. Another limitation of this study was that it was impossible to record the exact location and extent of demarcated opacities, enamel breakdowns, or atypical restorations in sufficient detail to allow conclusions to be drawn about FS retention behaviour. Notably, the survival of FSs on hypomineralized enamel might be reduced, but further studies are needed to investigate this hypothesis.

## Conclusion

On the basis of the documented data, FSs were frequently used among the Bavarian primary schoolchildren who participated in this study. No differences in sealant use were detected between individuals with or without MIH molars. Regardless of the presence of caries or MIH, the sealant quality was found to be satisfactory. With respect to the results from the mixed-effects logistic regression analysis, it was concluded that sealants are a caries-protective measure in primary schoolchildren with and without MIH. However, the caries-preventive effect decreased with increasing loss of sealant material.

## Data Availability

No datasets were generated or analysed during the current study.
